# Healthy Lifestyles Reduce the Incidence of Chronic Diseases and Dementia: Evidence from the Caerphilly Cohort Study

**DOI:** 10.1371/journal.pone.0081877

**Published:** 2013-12-09

**Authors:** Peter Elwood, Julieta Galante, Janet Pickering, Stephen Palmer, Antony Bayer, Yoav Ben-Shlomo, Marcus Longley, John Gallacher

**Affiliations:** 1 Cochrane Institute of Primary Care and Public Health, Cardiff University, Cardiff, United Kingdom; 2 School of Social and Community Medicine, University of Bristol, Bristol, United Kingdom; 3 Welsh Institute for Health and Social Care, University of South Wales, Pontypridd, United Kingdom; Emory University, United States of America

## Abstract

**Background:**

Healthy lifestyles based on non-smoking, an acceptable BMI, a high fruit and vegetable intake, regular physical activity, and low/moderate alcohol intake, are associated with reductions in the incidence of certain chronic diseases, but to date there is limited evidence on cognitive function and dementia.

**Methods:**

In 1979 healthy behaviours were recorded on 2,235 men aged 45–59 years in Caerphilly, UK. During the following 30 years incident diabetes, vascular disease, cancer and death were recorded, and in 2004 cognitive state was determined.

**Findings:**

Men who followed four or five of the behaviours had an odds ratio (OR) and confidence intervals (CI) for diabetes, corrected for age and social class, of 0.50 (95% CI: 0.19, 1.31; P for trend with increasing numbers of healthy behaviours <0.0005). For vascular disease the OR was 0.50 (95% CI: 0.30, 0.84; P for trend <0.0005), and there was a delay in vascular disease events of up to 12 years. Cancer incidence was not significantly related to lifestyle although there was a reduction associated with non-smoking (OR: 0.65; 95% CI: 0.54, 0.79). All-cause mortality was reduced in men following four or five behaviours (OR 0.40; 95% CI: 0.24, 0.67; P for trend <0.005).

After further adjustment for NART, the OR for men following four or five healthy behaviours was 0.36 (95% CI: 0.12, 1.09; P for trend <0.001) for cognitive impairment, and 0.36 (95% CI: 0.07, 1.99; P for trend <0.02) for dementia.

The adoption of a healthy lifestyle by men was low and appears not to have changed during the subsequent 30 years, with under 1% of men following all five of the behaviours and 5% reporting four or more in 1979 and in 2009.

**Interpretation:**

A healthy lifestyle is associated with increased disease-free survival and reduced cognitive impairment but the uptake remains low.

## Introduction

***‘And now let doctors quit the centre stage***

***To usher in the prophylactic age’***

From: ‘Superfluous Doctors’ in: Poems from a Prisoner of War Camp

AL Cochrane 1942

Lifestyle and health-related behaviours are powerful determinants of morbidity and mortality worldwide [1], [2], and unhealthy behaviours lie at the root of many chronic and disabling diseases [3]. Major concerns focus on smoking, body mass, physical activity, diet and alcohol consumption, and the concept of a healthy lifestyle is usually defined with reference to combinations of these factors. A number of studies have shown that the following of lifestyles based on these factors is strongly associated with reductions in the incidence of certain chronic diseases [4] [5] [6] [7]. Indeed, as one writer put it: ‘Healthy living is the best revenge’ [8].

Lifestyles, and in particular physical activity [9] [10] have been shown to be associated with cognitive health [11] [12]. Several reviews however have commented that the quality of the evidence is low and have called for more long-term cohort studies before conclusions can be drawn with confidence about the role of healthy behaviours in cognitive health [13], [14] [15]. A major issue is reverse causation. While this is a concern with all the outcomes, it is an especial concern in relation to dementia, because the pathophysiological processes associated with Alzheimer's Disease are known to begin many years prior to detectable cognitive impairment [16]. Evidence from short-term studies is therefore limited, seriously in the case of cognitive impairment, and long-term studies are greatly to be preferred.

The personal and public health benefits from healthy behaviours have been shown to have enormous potential so it is important that the uptake is carefully monitored. Evidence from a number of studies in the UK [4] [7] and elsewhere [6] [17] [5] show that the uptake of truly healthy lifestyles is very poor.

The Caerphilly Prospective Study (CaPS) is based on a cohort of men in a typical small town in South Wales UK. The men have been repeatedly questioned and examined for over 30 years. In this report we summarise evidence on relationships between healthy lifestyles at baseline and the incidence of diabetes, vascular disease, cancer, all-cause mortality, cognitive impairment and dementia during follow-up. We also examine changes in the following of healthy behaviours over the thirty years.

## Methods

Ethics approval was obtained from the South Wales Research Ethics Committee D, and each subject signed their agreement to be involved. Attempts were made to include in the cohort all men aged 45-59 years living in Caerphilly. Extensive data were collected at baseline (Phase I), including smoking history, self-reported physical activity and alcohol consumption, and, with help from his partner, each man completed a food frequency questionnaire. Extensive clinical data were also recorded at baseline. The present or most recent occupation of each man was used to derive social class as in the Office of National Statistics classification, and is summarised as ‘non-manual’ and ‘manual’.

The definitions of health related behaviours are based on answers to questions asked at baseline in 1979, but we have tried to match definitions used in recent studies, including the annual Welsh Health Survey [18], and other cohort studies [4] [6]. Thus, Smoking: men not smoking, including ex-smokers; body mass index (BMI): 18 to under 25 Kg/m2; Diet: consumption of fruit and vegetables was low in the community, therefore three or more portions of fruit and/or vegetables a day was accepted as ‘healthy’, together with less than 30% of calories from fat; Physical activity: walking two or more miles to work each day, or cycling ten or more miles to work each day, or ‘vigorous’ exercise described as a regular habit; Alcohol: three or fewer units per day, with abstinence not treated as a healthy behaviour.

The uptake of each of these behaviours, and combinations of them were later compared with similar data obtained in 2009 in the Welsh Health Survey [18], based upon self-reports from 15,000 adult subjects across Wales.

Approximately every five years after baseline the men were re-questioned and re-examined and primary care and hospital records inspected to identify new cases of diabetes type 2, and vascular events. Diabetes was self-reported. Clinical details of all possible ischaemic heart disease events, including electrocardiogram (ECG) and appropriate enzyme levels were evaluated against standard diagnostic criteria, and details of possible stroke symptoms together with computerized tomography (CT) scans were evaluated by two expert observers against standard diagnostic criteria. Notifications of deaths and cancer registrations were obtained from the Office of National Statistics.

In the second re-examination of the men, when they were aged 55–69 years, cognitive function testing was introduced into the study [19]. In 2004, when the survivors of the original cohort were aged 70–85 years, they were assessed in detail for cognitive impairment and for dementia, and the medical records of all the other men in the cohort were examined for evidence of cognitive impairment. Those who had a score on the Cambridge Cognitive Examination (CAMCOG) of less than 83, or a decline in CAMCOG score of 10 or more since the earlier cognitive examination, were selected for a clinical assessment. Full details of the clinical assessment of the selected men are reported elsewhere [20]. In brief: this included a modified CAMDEX interview [21]; the Rosen-revised Hachinski Ischaemic Score [22]; a neurological examination with Frontal Assessment Battery [23]; the Clinical Dementia Rating [24], and the Informant Questionnaire on Cognitive Decline in the Elderly [25].

### Statistical methods

Lifestyle was defined as the number of healthy behaviours practiced. Odds ratios (OR) and 95% confidence intervals (CI) were calculated for different numbers of healthy behaviours; number of behaviours was modeled as an ordinal variable. Men who practiced no healthy behaviours were used as the reference group. Trend was tested using an extension to the Wilcoxon Rank-Sum Test [26]. Adjustments were made for age and social class due to their strong relationships to all the outcomes and to the healthy behaviours. For cognitive impairment and dementia, additional adjustments were made for pre-morbid cognitive ability using the National Adult Reading Test (NART) [27] assessed at initial cognitive assessment (phase 3).

The main analyses compare incident cases with the remainder of the cohort. Sensitivity analyses were conducted by excluding men with evidence of disease at baseline (diabetes, a history of angina, chest pain, clinical or ECG evidence of infarction, stroke, high blood pressure). Reverse causation for cognitive outcomes was investigated by omitting men with evidence of early cognitive impairment: i.e. CAMCOG score monotonically declining from the initial cognitive assessment.

Rate advancement [28] gives an estimate of the average time by which any risk could, in theory, be postponed through the practice of a number of healthy behaviours. These times are calculated by comparing the effects of age on the outcomes with the effects of the healthy behaviours. A limitation in rate advancement is that it is only useful in the study of outcomes the rate of which increases with age. If the age relationship is weak, the rate advancement estimation is unreliable and not a useful measure. It is also worth noting that the estimates are conditional on the absence of competing risks. For example, if one disease is postponed, another disease may occur. Rate advancement could not be calculated for diabetes or dementia as no dates of onset were available.

## Results

At baseline, 2,235 men, representing 89% of the defined population, were examined. 46% were non-smokers and 35% had a BMI of 18 to under 25. Only fifteen men consumed five or more portions of fruit and/or vegetables daily, so the definition of this behaviour was reduced to three or more portions per day, and 18% of men satisfied this criterion. 39% took regular exercise, and 59% stated that they drank within the guidelines (Table 1).

**Table 1:** Odds ratios for individual healthy behaviours at base-line and the various outcomes during 30 years in the Caerphilly Prospective Study.

Healthy		Disease		Cognitive	function	Death
behaviours	Diabetes (214 men)	Vascular disease (752 men)	Cancer (648 men)	Any impairment (219) men)	Dementia (79) men)	(1,208 men)
**Non-smoking**	1.36	0.70	0.65	0.74	0.95	0.42
** **(1,017 men)	(1.02 to 1.82)	(0.58 to 0.84)	(0.54 to 0.79)	(0.50 to 1.08)	(0.54 to 1.69)	(0.35 to 0.51)
**BMI**: 18 to under 25	0.31	0.69	1.37	1.00	1.06	1.27
** **(791 men)	(0.21 to 0.45)	(0.57 to 0.83)	(1.13 to 1.66)	(0.66 to 1.51)	(0.58 to 1.94)	(1.05 to 1.53)
**Fruit/vegetable consumption 3+portions/day**	0.91	0.95	0.97	0.79	0.80	0.82
** **(394 men)	(0.62 to 1.33)	(0.75 to 1.21)	(0.76 to 1.23)	(0.52 to 1.20)	(0.40 to 1.61)	(0.65 to 1.03)
**Regular exercise**	0.63	0.89	1.06	0.62	0.41	0.82
** **(874 men)	(0.46 to 0.85)	(0.74 to 1.07)	(0.88 to 1.28)	(0.41 to 0.92)	(0.22 to 0.77)	(0.68 to 0.99)
**Alcohol intake** within limits	1.02	0.97	0.94	0.68	0.65	0.87
** **(1,320 men)	(0.77 to 1.37)	(0.81 to 1.16)	(0.78 to 1.14)	(0.46 to 1.00)	(0.37 to 1.16)	(0.72 to 1.04)

All odds ratios have been adjusted for age and social class.

[27]. Cognitive function has also been adjusted for the National Adult Reading Test

Vascular disease includes ischaemic heart disease and ischaemic stroke.

**Smoking**  =  men not smoking, including ex-smokers.

**BMI** (Body Mass Index)  =  18 to 25 Kg/m2.

**Fruit and vegetable consumption**  =  3 or more portions per day, plus less than 30% of calories from fat. The criterion for fruit and vegetable intake had to be reduced because only 15 men consumed five portions per day).

**Regular exercise**  =  walking two or more miles to work each day, or cycling ten or more miles to work each day, or ‘vigorous’ exercise described as a regular habit.

**Alcohol intake**  =  three or fewer units per day, with abstinence not treated as a healthy behaviour.

The numbers of men judged to be following a healthy lifestyle were as follows: 179 (8%) followed none of the five behaviours, 702 (31%) followed one behaviour, 814 (36%) followed two, 429 (19%) followed three, 111 (5%) followed four or five behaviours and only two (0.1%) followed all five behaviours (Table 2).

**Table 2:** Odds ratios (95% CI) for healthy lifestyles and cognitive impairment, dementia chronic diseases and all-cause death adjusted for age and social class for each outcome (number of incident events).

Healthy lifestyles (Number of behaviours)	Numbers (%) of men (Total 2,235)	Diabetes (214 men)	Vascular Disease (752 men)	Cancer (648 men)	Cognitive Impairment (219 men)	Dementia (79 men)	All-cause Deaths (1,208 men)
None	179 (8%)	1.00	1.00	1.00	1.00	1.00	1.00
		1.35	0.85	1.00	0.69	0.80	0.78
Any one	702 (31%)	(0.79–2.31)	(0.60–1.19)	(0.71–1.47)	(0.32–1.49)	(0.23–2.77)	(0.55–1.12)
		0.84	0.58	0.88	0.47	0.61	0.64
Any two	814 (36%)	(0.48–1.45)	(0.41–0.82)	(0.60–1.23)	(0.22–1.00)	(0.18–2.10)	(0.45–0.92)
		0.62	0.53	1.00	0.36	0.32	0.48
Any three	429 (19%)	(0.33–1.16)	(0.37–0.77)	(0.68–1.48)	(0.16–0.81)	(0.08–1.24)	(0.33–0.71)
		0.50	0.50	0.87	0.36	0.36	0.40
Four or five*	111 (5%)	(0.19–1.31)	(0.30–0.84)	(0.51–1.48)	(0.12–1.09)	(0.07–1.99)	(0.24–0.67)
Significance	of trend	<0.0005	<0.0005	0.60	0.001	0.02	<0.0005

* Data for the two men following all five behaviours have been included with those following four.

All the relationships have been adjusted for age and social class, and those for cognitive impairment and dementia have additionally been adjusted for NART (27) score at baseline.

Vascular disease includes ischaemic heart disease and ischaemic stroke.

The risk of diabetes declined with increasing numbers of healthy behaviours followed, up to an OR of 0.50 (95% CI: 0.19, 1.31, Table 2) with four or five behaviours, and the trend with increasing numbers of behaviours was significant (P<0.0005). For vascular disease the risk was decreased up to an OR of 0.50 (95% CI: 0.30, 0.84) with four or five behaviours, and again the trend was significant (P<0.0005). Cancer incidence was not related to lifestyle although there was a reduction in cancer associated with non-smoking alone (OR: 0.65; 95% CI 0.54, 0.79, Table 1). There was a significant association between lifestyle and all-cause mortality, with an OR of up to 0.40 (95% CI: 0.24, 0.67; P for trend <0.0005) with four or five behaviours.

Cognitive function was assessed in 1,225 men (75% of the survivors), and examination of the medical records of the other men identified a further 219 with evidence of impairment. Seventy-nine of these men were judged by two experienced clinicians to have dementia. After adjustment for age, social class and NART, the OR for cognitive impairment in men following four or five behaviours was 0.36 (95% CI: 0.12, 1.09; P for trend <0.001), and for dementia was 0.36 (95% CI: 0.07, 1.99; P for trend <0.02, Table 2). A detailed examination of relationships with individual behaviours suggested that exercise is an important predictor of both cognitive impairment (OR 0.64 95% CI 0.41, 0.92; P<0.04, Table 1) and dementia (OR 0.41 95% CI 0.22, 0.77; P<0.005).

A sensitivity analysis was conducted by omitting 896 men with evidence of diabetes or vascular disease at baseline. The association with all-cause mortality remained unchanged. In the men following four or five of the behaviours, the OR for diabetes decreased from 0.47 to 0.27 (95% CI: 0.06, 1.26; P for trend <0.002), and for vascular disease from 0.50 to 0.46 (95% CI: 0.22, 0.93; P for trend <0.0005).

A further sensitivity analysis was conducted with cognitive outcomes by omitting 144 men with evidence of early cognitive decline. This included 26 men with dementia. Due to the reduced numbers of men with dementia the upper two healthy behaviour groups were combined to form a three, four or five healthy behaviour group. For these men the OR for cognitive impairment was 0.48 (95% CI: 0.30, 0.79; P for trend <0.001) before omitting men with evidence of early impairment and 0.52 (95% CI: 0.30, 0.93; P for trend <0.001) after omitting men with evidence of early decline. For dementia, in men with between three and five healthy behaviours the OR was 0.40 (95% CI: 0.18, 0.86; P for trend <0.007) before omitting men with evidence of early impairment and 0.47 (95% CI: 0.18, 1.23; P for trend <0.05) after omitting men with evidence of early decline.

Over time there were changes in the uptake of the behaviours leading to a ‘dilution’ of the associations with disease. When questioned in the repeated follow-up examinations 1,023 men did not report substantial changes in the behaviours they followed. Amongst those who consistently reported either four or five healthy behaviours the trends with increasing numbers of behaviours remained significant. For diabetes the OR was 0.27 (95% CI: 0.04, 1.66; P for trend <0.002), for vascular disease the OR was 0.22 (95% CI: 0.05, 0.89; P for trend <0.0005), and for all-cause death the OR was 0.46 (95% CI: 0.14, 1.54; P for trend <0.001). For cognitive outcomes, due to small numbers 3–5 behaviours groups were collapsed, in which the OR for impairment was 0.82 (95% CI: 0.50, 1.36) and the OR for dementia was 0.75 (95% CI: 0.35, 1.61).

Rate advancement periods were estimated. For vascular disease, men following two, three and four healthy behaviours had delays of approximately 9.3, 10.8 and 11.9 years, respectively. For mortality the delays were 2.5, 4.6 and 6.3 years respectively.

Finally, the uptake of healthy behaviours and healthy lifestyles within the cohort in 1979 were compared with estimates of uptake in 2009 based on data from the Welsh Health Survey [18]. Individual healthy behaviour uptake changed over time: smoking declined, more fruits and vegetables were consumed, overweight increased and exercise declined. The adoption of a healthy lifestyle by men was however low and appears not to have changed during the subsequent 30 years, with under 1% of men following all five of the behaviours and 5% reporting four or more in 1979 and in 2009 (Table 3).

**Table 3:** Uptake of behaviours in the Caerphilly Prospective study and in the Welsh Health Survey [18].

Healthy behaviours in 1979	1979	2009	2009
	The Caerphilly cohort	The Welsh Health Survey	The Welsh Health Survey
	Number (%) of menAged 45–59 years	Number (%) of menaged 45–59 years	Number (%) of personsMale and female 16 +years
**Number of subjects**	2,235 men	1,927 men	15,810 persons
**Non smoking**	1,017 *(46%)*	1,405 *(73%)*	12,201 *(77%)*
**BMI** (18 to under 25)	791 *(35%)*	461 *(24%)*	6,027 *(38%)*
**Fruit & veg. consumption**	394 (18%)*	646 *(34%)*	5,431 *(34%)*
**Regular activity**	874 *(39%)*	680 *(35%)*	4,519 *(29%)*
**Light/Mod drinking**	1,320 *(59%)*	825 *(43%)*	8,638 *(55%)*
**Healthy Lifestyle**			
- **No healthy behaviours**	179 *(8%)*	135 *(7%)*	845 *(5%)*
- **Any two behaviours**	814 *(36%)*	650 *(34%)*	5,251 *(33%)*
- **Any three behaviours**	429 *(19%)*	350 *(18%)*	3,008 *(19%)*
- **Any four behaviours**	109 *(5%)*	98 *(5%)*	1,042 *(7%)*
- **All five behaviours**	2 (0.1%)	9 *(0*.*5%)*	132 *(0.8%)*

* The criterion for fruit and vegetable intake had to be reduced to 3 portions a day for the Caerphilly cohort because only 15 men in the 1979 cohort consumed five portions per day.

## Discussion

Within a representative sample of middle-aged men, the following of increasing numbers of healthy behaviours was associated with increasing reductions in several important chronic diseases and mortality: an estimated 50% reduction in diabetes, 50% in vascular disease and 60% for all-cause mortality. These results therefore confirm previous studies and provide further data on the association of lifestyle with cognitive impairment and dementia, with a reduction of about 60% in cognitive impairment and about the same in dementia. These reductions, and especially those in cognitive function, are of enormous importance in an ageing population.

When the ‘dilution’ effect of changes in lifestyle during follow-up is allowed for, the relationships we describe are closely similar to those reported from other cohorts. Thus the subjects amongst the 43,000 US Health Professionals who had adopted the five healthy behaviours experienced an 87% reduction in heart disease (Relative risk (RR): 0.13 CI: 0.09–0.19) [6] and among the 84,000 women in the US Nurses' Health Study the risk of coronary events was reduced 85% (RR: 0.17; 95% CI: 0.07–0.41) by following the five behaviours [5]. In another US study, a 61% reduction in type 2 diabetes was attributable to a low BMI alone [17] and this compares with 69% less diabetes in men in the CaPS with a BMI at baseline within the guidelines.

Similar effects have been reported in men and women in the European EPIC study: subjects following four healthy behaviours having approximately one-quarter the mortality of those who followed none (OR: 0.25; 95% CI: 0.18,0.34), equivalent to about 14 years difference in chronological age [4]. In a study of 4,886 adult British subjects, the following of four healthy behaviours led to hazard ratios of 0.29 (95% CI: 0.19, 0.43) for cardiovascular disease and 0.29 (95% CI: 0.19, 0.43) for death [7].

The absence of any reduction in cancer in CaPS, other than by non-smoking, is surprising. Other papers have shown reductions attributable to the other healthy behaviours, indeed in some the reduction is large, up to a hazard ratio of 0.30 (95% CI: 0.15, 0.60) [8]. On the whole however the reduction in cancer appears to be highly variable and usually small. For example, in 112,000 non-smoking subjects, the 4% of subjects who achieved a high score based on body weight, activity, diet and alcohol intake, showed a reduction of only 14% in incident cancer (RR: 0.86; 95% CI: 0.78, 0.94) during a follow-up of 14 years [29].

The postponement of vascular disease by healthy living for up to 12 years and up to six years for death is of interest, and again, these estimates are derived from subjects, all but two of whom followed four, rather than all five behaviours. A report based on two thousand 60 year old college alumni, estimated that the onset of ‘disability’ in eight common daily activities was delayed 8.3 years by following three healthy behaviours [30]. A study based on subjects within the US NHANES cohort who had adopted four healthy behaviours, estimated a rate advancement of 11.1 years for all-cause mortality [1], and a UK study of five thousand adults following four behaviours showed an all-cause mortality risk equivalent to being 12 years older than those following no healthy behaviour [7].

Of particular interest is the association of lifestyle with cognitive outcomes. Studies on elderly populations followed for short periods have generally shown an association between lifestyle and cognitive impairment [9] [31] [32] [33], but the likelihood of reverse causation makes the interpretation of such short –term studies difficult. In fact, few studies have followed middle-aged populations over extended periods. Within the Whitehall study obesity, alcohol and smoking have been show to affect cognitive function over 10 years [34], [35]. In the Honolulu-Asia Aging Study a healthy lifestyle was related to reduced risk of dementia over 25 years (OR 0.36, 95% CI 0.15, 0.84) [36]. Our data are consistent with these results.

The fact that the significant trends in our study for cognitive impairment and dementia were retained after omitting men with evidence of early cognitive impairment suggests that reverse causation is unlikely to contribute much to the relationships we describe. Although it can still be argued that even 30 years follow-up may not fully remove the full impact of cognitive change on behaviour, early pathological changes that have been described decades prior to the onset of dementia do not necessarily lead to cognitive change [37].

Reverse causation is unlikely to contribute much to the relationships with diabetes and vascular disease, or with mortality. These relationships remain significant following the omission of men with evidence of disease at baseline, and furthermore, omission of events during the first five years of follow-up was found to make no material difference to the associations (data not shown).

An important limitation in the CaPS study is the fact that the full impact of healthy lifestyles was underestimated because of small numbers of men adhering to all five healthy behaviours. In addition, the full effect of dilution due to changes in behaviour that were not fully monitored constantly throughout follow-up. A strength of CaPS however is that surveillance of the subjects in CaPS was close throughout the study, and intense efforts were made to identify incident disease involving both questioning of the subjects and inspection of all the available medical records approximately every five years.

These findings must be balanced against an earlier, more detailed analysis which, following extensive adjustment for health, social and other lifestyle factors, found no evidence for an independent association of physical activity with dementia [38]. This more detailed analysis suggests any benefit of physical activity on cognition is likely to be mediated via vascular metabolic and emotional pathways rather than being independent of these factors. While the independence of the relationship we describe can therefore be questioned, the public health message is still that regular physical activity is highly beneficial.

Residual confounding cannot be discounted in these data; however, the analyses we present investigate overall effects of lifestyle, rather than attempt to identify causal pathways between lifestyle and health. Non-causal explanations include confounding by social factors such as education and marital status. Nevertheless, our findings confirm that there is a substantial health benefit associated with a healthy lifestyle.

Writing about wellbeing in the community, Huppert [39] puts the case for a population approach, aiming for a small shift in an unhealthy behaviour, rather than focussing interventions on individuals who need help. Thus: assuming that the associations we report are causal and reversible, quite small increases in the uptake of healthy behaviours could considerably reduce the population burden of vascular disease, dementia and death. Had the two and a half thousand men in CaPS each been urged at baseline to adopt one additional healthy behaviour, and if only half them had complied, then during the following 30 years there would have been a 13% reduction in dementia, a 12% drop in diabetes, 6% less vascular disease and a 5% reduction in total mortality.

The area chosen for the CaPS cohort studies was selected because the social class distribution of the residents had been shown to be similar to that of the UK [40], and it can reasonably be assumed to be similar to that of the whole of Wales, a substantial part of the UK. Comparisons with the Welsh Health Survey show that there has been little change in the prevalence of healthy lifestyles during the 30 years covered by CaPS (Table 3). During this period it has been estimated that unhealthy living accounts for 10% of the costs of the National Health Service in Wales, while the annual expenditure on prevention and public health services in Wales has been estimated to have been around £ 280 millions (USD 436 millions) [41]. Despite this, and despite increasing knowledge of the relevance of lifestyle to health and to survival, the proportion of the adult Welsh population following all five healthy behaviours was, and remains under 1%. At the same time, the prevalence of all five healthy behaviours within health workers in the USA, Nurses [5] and other Health Professionals [6], is estimated to be only 3%. Clearly there is an urgent need for new strategies in health promotion to be developed and evaluated.

The costs of health services are increasing globally, and are likely to become unsustainable unless members of the public become more fully engaged and take a greater responsibility for their own health [42]. Personal prevention measures, such as we describe, could have a large impact on the costs of healthcare services [39]. Ultimately however, decisions about behaviours lie with the individuals and there is therefore an urgent need to establish a more effective partnership between health services and citizens.
